# Supplementary material for: Interactions between sensory prediction error and task error during implicit motor learning
Source: PLoS Comput Biol. 2022 Mar 23;18(3):e1010005. doi: 10.1371/journal.pcbi.1010005 (PMC8979451; doi:10.1371/journal.pcbi.1010005)
Supplement: S1 Text — (DOCX) [file pcbi.1010005.s001.docx]

**Similarities and differences between Experiments 1 and 2**

In both Experiments 1 and 2 (Fig 2,3), SPE + TE perturbations elicited robust implicit recalibration, whereas TE-only perturbations failed to elicit implicit recalibration. Interestingly, implicit recalibration in response to a 4° clamp was significantly greater in Exp 2 compared to Experiment 1 ($t_{\left( 30 \right)}=4.0, p<0.001, D= 1.5, [0.3, 0.8]$). Below, we provide three hypotheses that may have contributed to this difference between the two experiments.

First, visual errors are presented in a more variable manner in Exp 2 compared to Exp 1. That is, all trial types (SPE + TE, SPE-only, TE-only) were randomly presented throughout the experiment in Exp 2, whereas trial-types were presented in separate mini-blocks in Exp 1. Variable errors may increase the brain’s sensitivity to errors in an attempt to “keep up” with a more variable environment [1,2]. For instance, participants exhibited a faster learning rate in response to errors following a random walk schedule with errors drawn from a larger standard deviation and a slower learning rate in response to a random walk schedule with errors drawn from a smaller standard deviation. The effect of variability on error sensitivity may have resulted in increased adaptation in Exp 2, compared to Exp 1 (but see: [3,4]). Second, overall alertness may be enhanced when participants experience greater variability in the task environment. Greater attentiveness may in turn lead to an increase in the gain on error-based learning, and thus, increasing the rate of implicit recalibration in Exp 2 compared to Exp 1 [5,6].

Third, and lastly, given that participant’s in Exp 1 experience a wider range of errors (0° - 16°) compared to Exp 2 (0° - 4°), there may have been greater trial-by-trial interference in Exp 1. Specifically, ±16° SPE on trial n (a condition only in Exp 1) may result in significant anterograde interference [7,8].

Future work can directly test adjudicate among these hypotheses. For instance, errors can be drawn from different distributions and different sizes to modulate error variability. Periodic catch trials to rare target locations may serve as probes of general alertness. Nonetheless, in three different experimental contexts, there was a clear, replicated dissociation between SPE + TE and TE-only learning, strengthening the generalizability of our results.

**References**

1. Burge J, Ernst MO, Banks MS. The statistical determinants of adaptation rate in human reaching. J Vis. 2008 Apr 23;8(4):20.1-19.

2. Wei K, Körding K. Uncertainty of feedback and state estimation determines the speed of motor adaptation. Front Comput Neurosci. 2010 May 11;4:11.

3. Albert ST, Jang J, Sheahan HR, Teunissen L, Vandevoorde K, Herzfeld DJ, et al. An implicit memory of errors limits human sensorimotor adaptation. Nat Hum Behav [Internet]. 2021 Feb 4; Available from: http://dx.doi.org/10.1038/s41562-020-01036-x

4. Avraham G, Keizman M, Shmuelof L. Environmental consistency modulation of error sensitivity during motor adaptation is explicitly controlled. J Neurophysiol. 2020 Jan 1;123(1):57–69.

5. Wang TSL, Song J-H. Impaired visuomotor generalization by inconsistent attentional contexts. J Neurophysiol. 2017 Sep 1;118(3):1709–19.

6. Taylor JA, Thoroughman KA. Divided attention impairs human motor adaptation but not feedback control. J Neurophysiol. 2007 Jul;98(1):317–26.

7. Lerner G, Albert S, Caffaro PA, Villalta JI, Jacobacci F, Shadmehr R, et al. The Origins of Anterograde Interference in Visuomotor Adaptation. Cereb Cortex [Internet]. 2020 Mar 4; Available from: http://dx.doi.org/10.1093/cercor/bhaa016

8. Krakauer J, Ghez C, Ghilardi MF. Adaptation to visuomotor transformations: consolidation, interference, and forgetting. J Neurosci. 2005 Jan 12;25(2):473–8.
