# Supplementary material for: Interactions between sensory prediction error and task error during implicit motor learning
Source: PLoS Comput Biol. 2022 Mar 23;18(3):e1010005. doi: 10.1371/journal.pcbi.1010005 (PMC8979451; doi:10.1371/journal.pcbi.1010005)
Supplement: S2 Text — (DOCX) [file pcbi.1010005.s002.docx]

**Similarities and differences between in-person and online experiments**

Both in-lab (Exp 1A) and online results (Exp 1B), showed marked recalibration in response to SPE + TE perturbations but no recalibration in response to TE-only perturbations. However, while the magnitude of implicit recalibration was comparable across settings for ±16° in the SPE+TE condition, implicit adaptation was noticeably smaller in the online ±4° condition. In addition to these qualitatively similar results, there was also a correspondence between in-lab (Exp 2A, 2B) and online results (Exp 3, 4), where both types of experiments exhibited a marked asymmetrical effect of target jumps: Whereas jumping the target to the cursor attenuated recalibration, jumping the target away from the cursor resulted in similar levels of recalibration. Notably, this effect generalized across three different clamp sizes (3°, 4°, and 7°). However, the magnitude of the target jump effect differed across experiments. Specifically, jump-to in Exp 2 (4° clamp, in-person) yielded a 13% decrease in implicit adaptation, whereas jump-to in Exp 3 (3° clamp, online) yielded a 90% decrease. We do not have a ready explanation for these differences, but offer two possibilities.

First, the temporal resolution in stimuli presentation was better for the lab-based experiment with the KinArm compared to the online experiment in which participants used a trackpad controlled by a personal computer. The KinArm uses a feedforward algorithm to extrapolate the cursor’s trajectory during movement, resulting in minimal delay of the visual stimuli (trajectory feedback of the cursor) once the movement has begun. The standard computer, in contrast, does not use a feedforward algorithm, resulting in a delay of around 15 ms that varies with the browser and operating system [1]. There is also a small lag in the trackpad system in detecting the motion of the hand, which may have among many other reasons, resulted in smaller RTs and MTs (Table S1). Implicit sensorimotor recalibration is highly sensitive to the timing of feedback [2]; as such, any differences in stimulus timing may not only influence the degree to which the motor system recalibrates in response to errors but also the modulatory effect of target jumps.

Second, the experimental environment is more controlled in-person compared to online. Room lights, for example, are always extinguished in the laboratory to ensure that participants are readily perceive visual stimuli with high resolution. In addition, participants in the lab likely experience fewer environmental distractions. Participants recruited online, in contrast, are likely to complete the experiment in a well-lit room and be susceptible to distraction. These difference in lower-level visual perception (e.g., contrast, luminance of stimuli), as well as differences in general attention/alertness/motivation, may influence the degree of implicit adaptation and/or the distracting effect of target jumps, and thus account, at least in part for the small effect of target jumps in-person (13%) compared to online (90%). Consistent with increased distraction, participants in the online experiments exhibited greater hand angle variability in the baseline block compared to participants in the in-person experiments (Table S1).

Taken together, the differences in stimuli presentation time and experimental context may change how participants perceive and attend to the experiment, resulting in a greater influence of target jumps in the online experiments compared to those performed in-person. Importantly, despite the quantitative differences we have described here, the main results from the lab and online experiments were qualitatively consistent across different clamp sizes (3°, 4°, 7°), different experimental settings (online vs in-person), and different experimental contexts (mini-block vs interleaved).

**References**

1. Anwyl-Irvine A, Dalmaijer ES, Hodges N, Evershed JK. Realistic precision and accuracy of online experiment platforms, web browsers, and devices. Behav Res Methods [Internet]. 2020 Nov 2; Available from: http://dx.doi.org/10.3758/s13428-020-01501-5

2. Brudner SN, Kethidi N, Graeupner D, Ivry RB, Taylor JA. Delayed feedback during sensorimotor learning selectively disrupts adaptation but not strategy use. J Neurophysiol. 2016 Mar;115(3):1499–511.
