# Supplementary material for: Interactions between sensory prediction error and task error during implicit motor learning
Source: PLoS Comput Biol. 2022 Mar 23;18(3):e1010005. doi: 10.1371/journal.pcbi.1010005 (PMC8979451; doi:10.1371/journal.pcbi.1010005)
Supplement: S1 Table — (DOCX) [file pcbi.1010005.s003.docx]

**Table S1: A kinematic comparison across experiments.**

|  | **In-person** | | **Online** | | |
| --- | --- | --- | --- | --- | --- |
|  | **Exp 1A** | **Exp 2** | **Exp 1B** | **Exp 3** | **Exp 4** |
| **RT**  (ms) | 394.5  (35.9) | 395.8  (20.8) | 231.7  (11.1) | 312.0  (19.4) | 295.0  (11.0) |
| **MT**  (ms) | 329.1  (6.3) | 321.5  (3.6) | 175.1  (26.3) | 217.3  (32.2) | 140.8  (6.9) |
| **Hand angle SD**  (°) | 2.1  (0.1) | 2.3  (0.1) | 4.2  (1.8) | 4.6  (0.2) | 4.5  (0.1) |
