# Supplementary material for: Interactions between sensory prediction error and task error during implicit motor learning
Source: PLoS Comput Biol. 2022 Mar 23;18(3):e1010005. doi: 10.1371/journal.pcbi.1010005 (PMC8979451; doi:10.1371/journal.pcbi.1010005)
Supplement: S2 Table — (DOCX) [file pcbi.1010005.s004.docx]

**Table S2: TE-only fails to elicit implicit recalibration in response to small target displacements.** The mean slope value and its 95% confidence interval were computed using only target jumps between ±4° for Experiments 1 & 2A and ±8° for Experiment 2B.

| Experiment | Mean slope [95% CI] | P value |
| --- | --- | --- |
| 1A | -0.02 [-0.15 0.10] | 0.72 |
| 1B | -0.05 [-0.12 0.03] | 0.20 |
| 2A | 0.01 [-0.06 0.07] | 0.62 |
| 2B | -0.02 [-0.05 0.01] | 0.72 |
