# Supplementary material for: Interactions between sensory prediction error and task error during implicit motor learning
Source: PLoS Comput Biol. 2022 Mar 23;18(3):e1010005. doi: 10.1371/journal.pcbi.1010005 (PMC8979451; doi:10.1371/journal.pcbi.1010005)
Supplement: Attachment — Submitted filename: TJ_PlosComp_RevisionLetter_10Nov2021.docx [file pcbi.1010005.s005.docx]

**Revision PCOMPBIOL-D-21-01291 [EMID:0faa599e9fdebb0f].**

Dear Dr. Tsay,

Thank you very much for submitting your manuscript "Interactions between sensory prediction error and task error during implicit motor learning" for consideration at PLOS Computational Biology. As with all papers reviewed by the journal, your manuscript was reviewed by members of the editorial board and by several independent reviewers. The reviewers appreciated the attention to an important topic and the original experimental design. Based on the reviews, this is a borderline minor revision (with a major caveat) based on the reviewer comments we received raising concerns with the interpretation and consistency of some of the results. We are thus somewhat likely to accept this manuscript for publication, providing that you modify the manuscript according to the review recommendations and address the concerns of the reviewers (this *may* have to involve additional experiments if the reviewers points cannot be addressed by analysis alone). We would also appreciate if you could format the manuscript (perhaps a resubmission?) in the PLOS style when you respond, it will help a more fluent editorial and review process.

Please prepare and submit your revised manuscript within 30 days (the standard frame for minor revision). If you anticipate any delay, please let us know the expected resubmission date by replying to this email and we will be happy to grant you substantial more time if needed.

When you are ready to resubmit, please upload the following:

[1] A letter containing a detailed list of your responses to all review comments, and a description of the changes you have made in the manuscript. Please note while forming your response, if your article is accepted, you may have the opportunity to make the peer review history publicly available. The record will include editor decision letters (with reviews) and your responses to reviewer comments. If eligible, we will contact you to opt in or out

[2] Two versions of the revised manuscript: one with either highlights or tracked changes denoting where the text has been changed; the other a clean version (uploaded as the manuscript file).

Important additional instructions are given below your reviewer comments.

Thank you again for your submission to our journal. We hope that our editorial process has been constructive so far, and we welcome your feedback at any time. Please don't hesitate to contact us if you have any questions or comments.

Sincerely,

Aldo A Faisal

Associate Editor

PLOS Computational Biology

Wolfgang Einhäuser

Deputy Editor

PLOS Computational Biology

***********************

**Reviewer's Responses to Questions**

**Reviewer #1:** The study by Tsay and colleagues investigates how target error (TE) modulates implicit learning, which is based on sensory prediction error (SPE) during adaptation to visuomotor rotation. They used the trial-by-trial error clamp learning as the testbed and modulated the TE by “jumping” the target during the reaching movement. In this particular instantiation of both TE and SPE, they find that the hand deviation in the very next trial is modified when both SPE and TE are present, but not when only TE is present. In the former case, the target jump attenuates the implicit learning as a function of TE size. Also, the target jump with 0 TE also attenuates the learning, implying that distraction is the reason behind TE's influence. They also build several models to explain the influence of TE on SPE-based learning; the conclusion is that the target jump distracted people, thus attenuated learning.

The study consists of 4 experiments with a progressive exploration of the research questions. I find it technically sound with relatively clear data. The finding that TE alone could not drive implicit adaptation is timely in the field.

Thank you for this summary.

I have two major concerns: 1) the interpretation of the findings and 2) the seemingly conflicting results between experiments.

**R1.1:** The TE-only conditions basically provide a task-irrelevant zero-clamp cursor, which is assumed to make SPE zero. Then, the null effect of the target jump is observed in experiments 1 and 2. Hypothetically, if there is no cursor feedback shown during reaching, i.e., removing the zero-clamp manipulation, we would probably see the effect of the target jump. In this hypothetical experiment, the SPE is arguably zero or absent, but the TE starts to have its implicit learning effect. I raise this question to scrutinize the current interpretation of the absence of learning effect with a target jump + a zero-clamp cursor. Instead of claiming the TE would not lead to implicit learning, we could also interpret the findings as the zero-clamp helps participants ignore the target jump? In other words, the unusual combination of zero-clamp and target jump, while both are instructed to be ignored, leads to the absence of responses to target jump. Whether it can be generalized to a conclusion that pure TE would not cause any implicit learning shall be questioned.

This is a very interesting idea. We had assumed that the clamped feedback did not nullify a response to the task error because we saw an effect of TE (target jump) in conditions in which there was SPE + TE. We interpreted the absence of a TE response when SPE = 0 as a conditional effect: The TE response is dependent on the presence of SPE. But the reviewer points out that rather than think of the presence of SPE as a prerequisite, it may be that SPE = 0 is a special case that helps the participant ignore the target jump.

Following the reviewer’s suggestion, we ran the decisive experiment. We opted to conduct an online experiment (n=50), testing the hypothesis that the absence of a visual SPE would facilitate TE-based learning. (We note that we have previously validated online studies of sensorimotor adaptation, showing very similar results for experiments run on the online platform to those obtained in the lab (Tsay, Ivry, Lee, & Avraham, 2021)). There were two basic types of trials. 1) SPE+TE trials where the clamped feedback was presented at 0°, ±4°, or ±16° and the target remained stationary (thus SPE + TE). 2) TE-only trials where the target jumped 0°, ±4°, or ±16° and there was no visual feedback of hand position (SPE=0). Trials were randomized in a block of 200 perturbation trials (10/condition). Implicit recalibration was indexed by the trial-by-trial change in hand angle.

The results replicated what we reported in Exps 1 and 2 (Fig S1 in the revised manuscript, pasted below): SPE+TE elicited robust sign-dependent changes in hand angle whereas TE-only trials resulted in no detectable change in hand angle. Amongst the analyses we report, we flag here that the slope of the SPE+TE function was negative (Mean slope ± SEM: $\beta= -0.1\pm0.0; F_{\left( 1, 438 \right)}=160.4, p<0.001, \eta^{2}=0.1$), whereas the slope of the TE-only function was no different than 0 (Mean slope ± SEM: $\beta= 0.0\pm0.0; F_{\left( 1, 438 \right)}=0.9, p=0.35, \eta^{2}=0.0$).

These results provide additional evidence that TE by itself does not induce sensorimotor recalibration and rule out the hypothesis that a 0° clamp produces a disruptive effect on the mechanisms driving the response to task error. We are grateful to the reviewer for this suggestion, recognizing that the “disruption hypothesis” is quite reasonable.

We have opted to describe these data in the Results section (lines 134-144) but limit our presentation of the data to a new supplemental figure (Fig S1). We considered adding the functions to the figure panels in Fig 2. However, given the differences in methodology (online vs in-person, only partial overlap of conditions), we thought this might be misleading and thus opted to go with the supplemental figure.

**Figure S1:** **Task error alone does not elicit implicit recalibration (Exp S1).** Results from an online experiment to assess if TE alone would elicit adaptation in the absence of feedback correlated with hand position. **(a)** SPE + TE condition (top). The visual clamp followed a trajectory that was displaced from the target by 0°, ±4°, or ±16° and the target remained at the original position. As such SPE = TE. TE-only condition (bottom). The target jumped (0°, ±4°, or ±16°) immediately after movement initiation, introducing a TE. No cursor feedback was provided and thus there was no SPE. **(b)** Trial-by-trial change in hand angle for the SPE + TE trials (top) and TE-only trials (bottom). As such, there was no evidence of implicit recalibration even when the task error occurred without visual feedback.

**R1.2:** The discrepancies between experiments:
Why the same perturbations (say, +/-4 degree SPE+TE) produced different learning sizes in Exp1 and Exp2A, for example? See Figure 2c and 2e. Can it be attributed to the block vs. random design?

A post-hoc t-test on the 4° SPE + TE conditions confirms the reviewer’s intuitions: Participants in Experiment 2 (random design) adapted significantly more than participants in Exp 1 (blocked design) ($t_{\left( 30 \right)}=4.0, p<0.001, D= 1.5$). Three (non-exclusive) differences between the two experiments may have contributed to this difference in implicit recalibration:

1) Visual errors are presented in a more variable manner in Exp 2 compared to Exp 1. That is, all trial types (SPE + TE, SPE-only, TE-only) were randomly intermixed throughout Exp 2, whereas trial-types were presented in separate mini-blocks in Exp 1. Variable errors may increase the brain’s sensitivity to errors in an attempt to “keep up” with a more variable environment (Burge, Ernst, & Banks, 2008; Wei & Körding, 2010). Specifically, in the Burge study, participants exhibited a faster learning rate in response to errors following a random walk schedule and a slower learning rate in response to a constant perturbation magnitude. The effect of variability on error sensitivity may have resulted in increased adaptation in Exp 2, compared to Exp 1 (but see: (Albert et al., 2021; Avraham, Keizman, & Shmuelof, 2020)).

2) We suspect that alertness may be heightened when participants experience greater variability in the task environment (Exp 2). Greater attentiveness may increase the gain on error-based learning (Taylor & Thoroughman, 2007; Wang & Song, 2017), and thus, increase the rate of implicit recalibration in Exp 2 compared to Exp 1.

3) Participants in Exp 1 experienced a wider range of errors (-16° to +16°) compared to Exp 2 (-4° to +4°) and this may have produced greater interference between trials in Exp 1, resulting in attenuated implicit recalibration (Avraham, Ryan Morehead, Kim, & Ivry, 2021). Specifically, a -16° SPE on trial n may have greater anterograde interference on trial n+1 compared to a -4° SPE on trial n (Krakauer, Ghez, & Ghilardi, 2005; Lerner et al., 2020).

We recognize that point #3 is in opposition to our first conjecture regarding variable errors. Future experiments could be designed to evaluate these hypotheses. For instance, errors can be drawn from different distributions and different sizes to modulate error variability. Periodic catch trials to rare target locations could serve as probes of general alertness. Nonetheless, in two different experiments, there was a clear, replicated dissociation between SPE + TE and TE-only learning, strengthening the generalizability of our results.

We now include a new supplemental section, titled “Similarities and differences between Experiments 1 and 2”, in which we discuss these issues.

**R1.3**: Exp2A-B and Exp 3A also differed quite a bit (figure 4). If we assume the 3-degree and 4-degree error clamp adaptation is similar (as they should be), we would expect to see a similar learning modulation of recalibration by the target jump. Thus, the modulation shall be linear, as shown in Exp3A and 3B and in the proposed Distracted SPE model. However, we observed a few obvious differences between the two experiments. For example, for a jump to -4 degree, Exp2 still produced large learning amounting to about 1 degree for an effective TE of size 0; in contrast, for a jump to -3 degree, Exp3 produced a nearly zero learning for an effective TE of size 0. How to reconcile the considerable difference between these two experiments, besides that Exp2 was performed in person and Exp3 was performed online?

The reviewer correctly points out that jump-to in Exp 2 (4° clamp, in-person) yielded a 13% decrease in implicit adaptation, whereas jump-to in Exp 3 (3° clamp, online) yielded a 90% decrease. We do not have a ready explanation for this difference, but offer two possibilities related to the two different testing platforms that were used in this study:

1) The temporal resolution in stimuli presentation was better in-person with the KinArm compared to online with the standard laptop. Specifically, the KinArm uses a feedforward algorithm to extrapolate the cursor’s trajectory during movement which results in minimal delay in visual stimuli (trajectory feedback of the cursor) once the movement has begun. The standard laptop computer, in contrast, does not use any feedforward algorithm, resulting in a 15 ms average delay that varies with the browser and operating system (Anwyl-Irvine, Dalmaijer, Hodges, & Evershed, 2020; Bridges, Pitiot, MacAskill, & Peirce, 2020). Implicit sensorimotor recalibration is highly sensitive to the timing of feedback (Brudner, Kethidi, Graeupner, Ivry, & Taylor, 2016; Hadjiosif et al., 2014; Kitazawa, Kimura, & Uka, 1997), and as such, any differences in stimulus timing may not only influence the degree in which the motor system recalibrates in response to errors but also the modulatory effect of target jumps.

2) The experimental environment is more controlled in-person compared to online. Room lights, for example, are always extinguished in the laboratory to ensure that participants are always able to perceive visual stimuli with high resolution. In addition, participants in the lab are likely more focused on the experiment. Participants recruited online, in contrast, are more likely to complete the experiment in a well-lit room, and as such, the contrast between visual stimuli is more variable. In addition, online participants may be more susceptible to distraction. These difference in lower-level visual perception (e.g., contrast, luminance of stimuli), as well as differences in general attention/alertness/motivation, may influence how susceptible participants are to the distracting effect of target jumps, leading to a smaller effect of target jumps in-person (13%) compared to the larger effects seen online (90%).

Taken together, the differences in stimuli presentation time and experimental context may change how participants perceive and attend to the experiment, resulting in a greater influence of target jumps in the online experiments compared to those performed in-person. Nonetheless, even though the magnitude of the effect differed across experiments, the qualitative effect of target jumps was consistent across different clamp sizes (3°, 4°, 7°), different experimental settings (online vs in-person), and different experimental contexts (mini-block vs interleaved).

We have now included a new Supplemental materials section, titled “Similarities and differences between in-person and online experiments”, detailing these hypotheses.

**Minor issues: Issues with the description of the experimental design:**

**R1.4:** Line 124: it is unclear to me how the authors examined the learning without providing TE-only and SPE+TE trials in separate blocks. Exp1 essentially has a mini-block design.

There were two types of trials: TE-only and SPE + TE trials. In Experiment 1, TE-only trials and SPE + TE trials were provided in *separate* mini-blocks, whereas in Experiment 2 these two trial types were randomized across the entire experiment. We have clarified this in the Results (lines 124 - 132) and Methods (lines 486 - 496) sections of the manuscript.

**R1.5:** Line462: what is the so-called random, zero-mean order? Five conditions per mini-block? Are conditions randomized across trials within a mini-block? It is unclear how the experiment trials are organized for these two experiments.

Please refer to R1.4. The sequence of trial types was pseudo-randomized by constraining the mean error to 0° over 20 consecutive trials in Exp 1 and 24 trials in Exp 2. This “zero-mean” constraint was to prevent any systematic drifts in hand angle towards one direction. Specifically, the measure of trial-by-trial recalibration would be a more “pure” measure of motor update, with minimal influence of retention/decay back to the target as described by state space models of adaptation (Donchin, Francis, & Shadmehr, 2003; Jordan & Rumelhart, 1992).

**R1.6**: Figures 2a and 2b give the impression that the trial after a perturbation (trial n+1) is a catch trial with SPE=0 and TE=0. But this is not the case, right? Then, how can we evaluate the learning in the next trial?

We now realize that Figures 2a and 2b were misleading: There were no catch trials, but instead, the order of trial types (SPE + TE, TE-only) was pseudo-randomized by constraining the mean error to 0° over every 20 consecutive trials in Exp 1 and 24 trials in Exp 2 (See R1.4; R1.5). In the figures, we chose to show a 0° clamp on trial n+1 to highlight the adaptive response. We reasoned that depicting another random perturbation on trial n+1 may confuse the reader to consider the significance on this perturbation as opposed to the behavioral response to the perturbation on trial n.

Implicit recalibration was defined as the resultant change in hand angle from trial n to trial n +1, as a function of the error provided on trial n. This measure of trial-by-trial recalibration has been widely used in previous literature (Avraham et al., 2020; Tsay et al., 2020; Vandevoorde & Orban de Xivry, 2019; Wei & Körding, 2009, 2010). The results of trial-by-trial recalibration are similar to those observed in other designs (e.g., see similar saturated motor update functions in Hayashi et al, who used a catch trial design, and Wei et al, who used a trial-by-trial design) (Hayashi, Kato, & Nozaki, 2020; Wei & Körding, 2009). An overall advantage of this method is that more perturbation trials can be obtained within a block compared to when each perturbation trial is surrounded by null trials.

We have now updated the Figure 2 caption, noting that the TE-only trial illustrated for trial n+1 was selected for simplicity in exposition. We have also included relevant references that have used the trial-by-trial measure of implicit recalibration in the Methods section (lines 542 - 544).

Line 157: less than “twice” the magnitude of the cursor perturbation??

This phrase/parenthetical has now been deleted.

**R1.7:** Line166: the citation format shall be checked throughout. Here a full name is used.

Modified.

**R1.8:** Line181: In to in

Modified.

**R1.9:** Figure 4: it might be better to show the linear trend in a and c by connecting the dots representing “jump-in-place 0 deg” condition. Thus all three data points are for conditions with a jumping target.

We appreciate the reviewer’s reasoning about why connecting the dots can highlight the linear effect of jumping the target. We nonetheless also want to emphasize the striking *asymmetry* associated with jumping the target: Specifically, jumping the target to the cursor attenuated implicit recalibration relative to no-jump, whereas jumping the target away from the cursor led to a relatively invariant change in behavior. This asymmetry is only predicted by the Distracted SPE + TE model (the best fitting model), and the current format makes this point most salient. We thought that adding an additional line to connect the target jump conditions would make the figured crowded, and as such, we’ve opted to keep the original figures.

**R1.10:** Line241: the findings did not provide direct support for the statement that the target jump modulates the size of the TE. In fact, we assume the target jump affects TE size, and we observe that implicit recalibration is modified as a result (besides the distraction effect).

Indeed, we can only *infer* that implicit recalibration is impacted by the strength of TE, which itself is a function of the size of the target jump (Distracted SPE + TE model). These changes are now reflected in lines 256 - 258.

**R1.11:** Line261: the slope of the TE function is about 0.02. Does it also hold for Exp1 where a larger target jump is used?

In Experiment 1, we compared implicit recalibration in response to SPE + TE vs TE-only conditions. We did not find that TE-only elicited implicit recalibration: The mean slope was zero (Figure 2d; $\beta= 0.0\pm0.0; t_{\left( 212 \right)}=0.6, p=0.69, D=0.1$).

**R1.12:** Line270 and Line272: the numbers are all approximate; please state it clearly.

We now state the exact numbers in lines 278 - 283.

**R1.13:** Line 271: The real test of the model should be testing its predictions with fitted parameters. For example, the winning model, the Distracted SPE+TE model, predicts that the SPE learning shall be effectively zero with a target jump larger than 35 degrees. This is a strong prediction, which can be easily tested. Currently, the model comparisons are about model fitting without rigorous testing.

Our primary objective in formulating the models was to quantitatively compare a variety of theories about potential ways in which cursor clamps and target jumps might interact to shape learning. Our goal was to formulate a series of models that encapsulated a range of qualitatively distinct effects, which we could then dissociate through model comparison. Our goal was not necessarily to devise a “definitive” model that can make quantitatively precise predictions for all possible experimental settings. We expect that our models may be falsified by more extensive datasets, and be bettered by more refined formulations. Nevertheless, we believe that our models do provide a good characterization of participants behavior, accounting for a decent amount of variance in the data (Distracted SPE + TE model R^2^ = 0.72) relative to other models (next best model R^2^ = 0.53). Together, these modeling results provide a sound basis for dissociating between the various underlying hypotheses we considered.

We agree that a general challenge for any proposed model is to assess model performance across as many novel experimental contexts as possible. For that reason, we have embedded several tests of generality (especially Exp 3 and 4) across the experiments. For instance, the asymmetrical effect of target jumps on implicit recalibration seen in Exp 3 applied to *both* the 3° and 7° clamped feedback groups. This asymmetrical effect was only predicted by the Distracted SPE + TE model.

More critically, and consistent with the line of reasoning of the reviewer, we wanted to stress test our winning model to see whether implicit adaptation follows an asymmetrical pattern over a wider range of target jumps. If this were the case, then implicit recalibration would diminish towards 0° when the target jumps in the direction towards, and past, the cursor. Implicit adaptation would be largely maintained when the target jumps away. These predictions were confirmed not only qualitatively, but also quantitatively in Exp 4 in both 3° and 7° clamped feedback groups (via R^2^ and AIC scores).

There is nonetheless more work to do to investigate the precise effect of TE, especially since we were not able to elicit any recalibration from target jumps alone, and therefore, were unable to detect any isolated effect of TE (Exp 1 and 2). Along the same lines, the effect of TE in the presence of SPE may not be linear – this was a simplifying assumption on our part in an effort to be parsimonious. Instead, the effect of TE may be non-linear for an even wider range of target jumps. Future studies that use different manipulations to vary TE without requiring target jumps (e.g., a positive tone, score, monetary reward) may shed more light on the constraints underlying TE’s modulatory effect on implicit recalibration.

**R1.14:** Table 2: negative R squares?

Negative values are possible when using the standard equation for R^2^: 1 - (sum of squared errors of our model) / (sum of squared errors from the null model (i.e., the mean of the data points)). As such, a negative R^2^ implies that the best fitting model performed worse than simply calculating the mean of the data points. We have made this definition of R^2^ explicit in lines 590 - 592.

**Reviewer #2:** In the article “Interactions between sensory prediction error and task error during implicit motor learning,” the authors look to determine the influence/interaction between different error signals on implicit changes in movement. Participants reached to targets while the sensory prediction error (visual clamp) and task error (target jump) were manipulated. Results indicated that task errors failed to lead to implicit changes in reaches in the absence of a sensory prediction error. However, when a sensory prediction error was present, task error modulated implicit changes in reaches. As well implicit changes in reaches were modified even if the target simply flickered in place. The authors went on to look to model their results using 3 different computational models. Results were best accounted for by a computational model in which sensory prediction errors and task errors contribute to implicit changes in reaches, and this contribution is modulated by attention.

The paper poses an interesting and timely question, as currently there is much work being done to discover the processes underlying implicit and explicit motor adaptation. The inclusion of the modelling work strengthens the paper and provides direction for future work. Below I outline a few questions/comments.

Thank you for the encouraging statements.

**R2.1:** The paper does not follow the typical organization of articles in PLOS, including the methods at the end of the manuscript. I assume this needs to be altered moving forward, and may make the manuscript easier to follow. As well, the reference to figures and panels in figures is not always correct (e.g., see Figure 3 caption).

We have updated the format of our article to fit the PLOS format. The materials and methods were kept at the end, based on the PLOS guidelines: <https://journals.plos.org/ploscompbiol/s/submission-guidelines>. We have also fixed the captions for Figure 3 – thank you for pointing out this error.

**R2.2:** The authors speak of the work providing insight into motor learning/adaptation (though the word recalibration is also used). In the current paradigm, error signals change from trial-to-trial. Thus, it is unclear if results can provide insight into motor learning, as there are no long term changes in behaviour. The authors should make clear how these results can/cannot speak to previous findings in visuomotor adaptation literature, which traditionally consider implicit adaptation as reflecting permanent changes in reach performance (i.e., aftereffects) that arise slowly.

We borrowed our terminology from a recent review article by Krakauer and colleagues (Krakauer, Hadjiosif, Xu, Wong, & Haith, 2019). There the authors provide a two-part definition of motor learning, the second part being “the ability to maintain performance levels of existing skills under changing conditions”, a direct reference to motor adaptation. We raise this point here to clarify that in our thinking adaptation is a critical component of motor learning. Furthermore, the review highlights recent work showing that motor “adaptation” tasks generally include at least two processes: Implicit recalibration (or implicit adaptation) and explicit aiming. We now include a short statement in the Introduction, making clear that “implicit recalibration” and “implicit adaptation” have historically been used interchangeably: Lines 47.

Implicit recalibration is typically measured in two ways. As the reviewer noted, implicit recalibration can be measured in a blocked design with a constant perturbation. Implicit adaptation thus accumulates and results in a marked aftereffect. These aftereffects are constrained by how much the motor system learns from the error *and* how fast the motor system “forgets”. State-space models provide one tool to estimate the parameters associated with these processes. Forgetting can also be empirically measured by how fast an aftereffect decays when the error signal is removed (Kitago et al, 2013).

Alternatively, implicit recalibration can also be measured using a trial-by-trial design, in which the error size and direction varies across trials (Avraham et al., 2020; Hutter & Taylor, 2018; Tsay et al., 2020; Wei & Körding, 2009, 2010). Typically, as in our experiment, the errors are scheduled in a zero-mean manner (see R1.4-6 for more details) such that hand angles remain in the vicinity of the target. Consequently, the effect of forgetting on changes in hand angle is minimal, with the resultant behavior being a “purer” measure of trial-by-trial motor updating. This design helps us examine whether the motor system is sensitive to TE and target jumps, with the focus being more on motor updating and less on retention.

There is a close correspondence between our trial-by-trial results with those obtained from blocked designs. Specifically, our finding that TE-only (target jump only) perturbations do not elicit trial-by-trial implicit recalibration is in accord with results reported in recent preprint that used a blocked design (Oza, Kumar, & Mutha, 2020): As shown in a key figure from their preprint below, no implicit recalibration was elicited by a constant 10° and 20° target jump. Moreover, the attenuating effects of jump-to in Exp 2-4 of our study were also seen in the blocked design studies reported in Leow et al (Leow, Marinovic, de Rugy, & Carroll, 2018, 2020). Taken together, the evidence indicates that properties of implicit recalibration can be identified in both trial-by-trial and blocked designs. Future work could test whether TE and target jumps influence retention using a blocked design with a long block of no-feedback aftereffect trials.

We have now several sentences to the Discussion, stating that our findings using a trial-by-trial design have been corroborated by those discovered via a Blocked design (lines 322 - 326).

**R2.3:** The introduction of the target jump and the resulting target error signal is an interesting manipulation. Previous work using target jumps, has suggested that the hand is automatically drawn to the new target position unconsciously (e.g., Goodale and Milner’s dual visual systems model; Pisella and colleagues’ work on the automatic pilot). How does the potential for the hand to be automatically updated in response to a target jump (even when it is not supposed to move in the direction of the target jump) influence the current results and resulting interpretation?

The work of Goodale and Milner and Pisella’s group has highlighted how movements are modified online in response to target jumps on the *current* trial. In our study, we are looking at how perturbations, either to the cursor or target influence behavior on the *next* trial.

We surmise that the key difference between these studies is the emphasis on either feedback control or feedforward control. In their studies, participants make an initial eye or hand movement towards the original target location, but unbeknownst to them, the target jumps, which results in an online, automatic corrective response towards the new target location. In contrast, our studies examine the effect of target jumps on feedforward corrections, measuring how participants update their sensorimotor map such that the same movement cue (the original target) would elicit a more accurate movement when that context is encountered in the future. We discouraged feedback corrections by requiring participants to make a ballistic movement *through* the target, and by imposing strict movement velocity/time constraints (40 cm/s - 70 cm/s at peak velocity for in-person experiments; less than 300 ms for online experiments). Moreover, there were no qualitative differences in the results when we used hand angles at peak velocity as a dependent variable (initial heading angle), instead of hand angle at the endpoint position, providing direct evidence that our results were not influenced by feedback control.

Feedback and feedforward systems seem to rely on different processes and different neural circuitries, with the former relying more on cortex whereas the latter relies more on the cerebellar circuitry in subcortex. For instance, patients with cerebellar lesions do not show deficits in online feedback control, but show striking deficits in feedforward control (Parrell, Agnew, Nagarajan, Houde, & Ivry, 2017; Parrell, Kim, Breska, Saxena, & Ivry, 2021; Zimmet, Cao, Bastian, & Cowan, 2020). We now include a paragraph in the Methods section underscoring how we depart from these classic studies in studying how feedforward motor recalibration is affected by task errors/target jumps (lines 422 - 438).

**R2.4:** The authors speak to attention/distraction as having an influence on implicit recalibration. How does the availability of the sensory prediction error influence results? For example, the stationary target jump/flicker would have decreased the time that participants experienced the sensory prediction error (potentially giving rise the fixed cost observed). Alternatively, could other factors arising as a result of change in paradigm influence performance (i.e., not just attention)?

As the reviewer notes, by transiently removing the original target via a target jump/flicker, the error signal may be less available (flicker: one sample of the original target removed; jump: multiple samples of the original target removed), resulting in attenuated implicit recalibration. This hypothesis, however, is difficult to reconcile with previous studies using more extreme manipulations of SPE strength. For instance, while implicit recalibration is attenuated in response to endpoint feedback compared to online trajectory feedback, the implicit response is still quite pronounced in the former condition (Taylor, Krakauer, & Ivry, 2014). Moreover, participants demonstrate robust implicit recalibration during conditions in which visual feedback of the target is removed for the entire duration of the reach (target disappears prior to movement onset: (Tsay et al., 2020)).

We hypothesize that the way in which a sensory prediction error is processed may relate to how the brain processes the visual input from the cursor. Specifically, more attentional resources given to the visual cursor may result in a greater gain on the sensory prediction error. Any changes in the original target location (flicker) may transiently remove attention away from the cursor, and therefore, decrease the strength of the cursor/SPE, attenuating implicit recalibration. This is how we frame things in the manuscript.

We now mention different ways in which target jumps might influence an SPE in the Results section to motivate the Distracted SPE and Distracted SPE + TE models (Lines 184 - 188).

**R2.5:** In the current study, 2 experiments were conducted online. The general data trends look similar to the results presented for the first 2 in-person experiments. That said, I am curious what the data looks like with respect to RT, MT, variability in performance measures, etc. Given the lack of published data related to online experiments, it would be beneficial to have sample data available.

The close correspondence between in-lab (Exp 1-2) and online results (Exp 3-4) is encouraging. Validating the online experiment was a priority for us, since as the reviewer pointed out, there is a paucity of motor learning studies performed online. Indeed, that is exactly what motivated our recent paper validating our online platform for studying sensorimotor learning. In that paper, we show a close correspondence between three classic visuomotor learning experiments performed in-person and online (Tsay et al., 2021).

The table below compares RT, MT, and hand angle variability (standard deviation of hand angles during feedback baseline) between the two experimental settings used in the current study. Overall, RT and MT are faster in the online setting, and hand angle variability is greater online (a finding corroborated by Tsay et al, 2021). There are many factors that could underlie these differences. The target distance is greater in the in-person setting (10 cm) compared to the online setting (6 cm). This is likely to not only impact MT, but also RT (Munro, Plumb, Wilson, Williams, & Mon-Williams, 2007). However, it is important to emphasize that the movements are performed very differently: In-person reaches require intersegmental control of the entire arm whereas online movements likely have some involvement of the wrist and fingers, and inertial factors associated with these different effectors will impact RT and MT (Anson, 1989). As such, the comparison of kinematics between the two settings is a bit of an apples and oranges comparison. We actually look at this as a glass half full: Despite these kinematic and neuromechanical differences, the results in terms of adaptation are quite similar across the two settings.

|  | In-person | | Online | |
| --- | --- | --- | --- | --- |
|  | Exp 1 | Exp 2 | Exp 3 | Exp 4 |
| RT | 394.5  (35.9) | 395.8  (20.8) | 312.0  (19.4) | 295.0  (11.0) |
| MT | 329.1  (6.3) | 321.5  (3.6) | 217.3  (32.2) | 140.8  (6.9) |
| Detrended  Hand angle  SD | 2.1  (0.1) | 2.3  (0.1) | 4.6  (0.2) | 4.5  (0.1) |

We have now included a new Supplemental materials section, titled “Similarities and differences between in-person and online experiments”.

**References**

Albert, S. T., Jang, J., Sheahan, H. R., Teunissen, L., Vandevoorde, K., Herzfeld, D. J., & Shadmehr, R. (2021). An implicit memory of errors limits human sensorimotor adaptation. *Nature Human Behaviour*. doi:10.1038/s41562-020-01036-x

Anson, J. G. (1989). Effects of moment of inertia on simple reaction time. *Journal of Motor Behavior*, *21*(1), 60–71.

Anwyl-Irvine, A. L., Dalmaijer, E. S., Hodges, N., & Evershed, J. (2020). *Online Timing Accuracy and Precision: A comparison of platforms, browsers, and participant’s devices*. doi:10.31234/osf.io/jfeca

Avraham, G., Keizman, M., & Shmuelof, L. (2020). Environmental consistency modulation of error sensitivity during motor adaptation is explicitly controlled. *Journal of Neurophysiology*, *123*(1), 57–69.

Avraham, G., Ryan Morehead, J., Kim, H. E., & Ivry, R. B. (2021). Reexposure to a sensorimotor perturbation produces opposite effects on explicit and implicit learning processes. *PLoS Biology*, *19*(3), e3001147.

Bridges, D., Pitiot, A., MacAskill, M. R., & Peirce, J. W. (2020). The timing mega-study: comparing a range of experiment generators, both lab-based and online. *PeerJ*, *8*, e9414.

Brudner, S. N., Kethidi, N., Graeupner, D., Ivry, R. B., & Taylor, J. A. (2016). Delayed feedback during sensorimotor learning selectively disrupts adaptation but not strategy use. *Journal of Neurophysiology*, *115*(3), 1499–1511.

Burge, J., Ernst, M. O., & Banks, M. S. (2008). The statistical determinants of adaptation rate in human reaching. *Journal of Vision*, *8*(4), 20.1-19.

Donchin, O., Francis, J. T., & Shadmehr, R. (2003). Quantifying generalization from trial-by-trial behavior of adaptive systems that learn with basis functions: theory and experiments in human motor control. *The Journal of Neuroscience: The Official Journal of the Society for Neuroscience*, *23*(27), 9032–9045.

Hadjiosif, A. M., Criscimagna-Hemminger, S. E., Gibo, T. L., Okamura, A. M., Shadmehr, R., Bastian, A. J., & Smith, M. A. (2014). Cerebellar damage reduces the stability of motor memories. *Proceeding of the Translational and Computational Motor Control*.

Hayashi, T., Kato, Y., & Nozaki, D. (2020). Divisively Normalized Integration of Multisensory Error Information Develops Motor Memories Specific to Vision and Proprioception. *The Journal of Neuroscience: The Official Journal of the Society for Neuroscience*, *40*(7), 1560–1570.

Hutter, S. A., & Taylor, J. A. (2018). Relative sensitivity of explicit reaiming and implicit motor adaptation. *Journal of Neurophysiology*, *120*(5), 2640–2648.

Jordan, M. I., & Rumelhart, D. E. (1992). Forward models: Supervised learning with a distal teacher. *Cognitive Science*, *16*(3), 307–354.

Kitazawa, S., Kimura, T., & Uka, T. (1997). Prism adaptation of reaching movements: specificity for the velocity of reaching. *The Journal of Neuroscience: The Official Journal of the Society for Neuroscience*, *17*(4), 1481–1492.

Krakauer, J., Ghez, C., & Ghilardi, M. F. (2005). Adaptation to visuomotor transformations: consolidation, interference, and forgetting. *The Journal of Neuroscience: The Official Journal of the Society for Neuroscience*, *25*(2), 473–478.

Krakauer, J., Hadjiosif, A. M., Xu, J., Wong, A. L., & Haith, A. M. (2019). Motor Learning. *Comprehensive Physiology*, *9*(2), 613–663.

Leow, L.-A., Marinovic, W., de Rugy, A., & Carroll, T. J. (2018). Task errors contribute to implicit aftereffects in sensorimotor adaptation. *The European Journal of Neuroscience*, *48*(11), 3397–3409.

Leow, L.-A., Marinovic, W., de Rugy, A., & Carroll, T. J. (2020). Task Errors Drive Memories That Improve Sensorimotor Adaptation (pp. 3075–3088). doi:10.1523/JNEUROSCI.1506-19.2020

Lerner, G., Albert, S., Caffaro, P. A., Villalta, J. I., Jacobacci, F., Shadmehr, R., & Della-Maggiore, V. (2020). The Origins of Anterograde Interference in Visuomotor Adaptation. *Cerebral Cortex* . doi:10.1093/cercor/bhaa016

Munro, H., Plumb, M. S., Wilson, A. D., Williams, J. H. G., & Mon-Williams, M. (2007). The effect of distance on reaction time in aiming movements. *Experimental Brain Research. Experimentelle Hirnforschung. Experimentation Cerebrale*, *183*(2), 249–257.

Oza, A., Kumar, A., & Mutha, P. K. (2020). Task Errors Do Not Induce Implicit Sensorimotor Learning (p. 2020.11.13.381285). doi:10.1101/2020.11.13.381285

Parrell, B., Agnew, Z., Nagarajan, S., Houde, J., & Ivry, R. B. (2017). Impaired Feedforward Control and Enhanced Feedback Control of Speech in Patients with Cerebellar Degeneration. *The Journal of Neuroscience: The Official Journal of the Society for Neuroscience*, *37*(38), 9249–9258.

Parrell, B., Kim, H. E., Breska, A., Saxena, A., & Ivry, R. B. (2021). Differential effects of cerebellar degeneration on feedforward versus feedback control across speech and reaching movements. *The Journal of Neuroscience: The Official Journal of the Society for Neuroscience*. doi:10.1523/JNEUROSCI.0739-21.2021

Taylor, J. A., Krakauer, J. W., & Ivry, R. B. (2014). Explicit and implicit contributions to learning in a sensorimotor adaptation task. *The Journal of Neuroscience: The Official Journal of the Society for Neuroscience*, *34*(8), 3023–3032.

Taylor, J. A., & Thoroughman, K. A. (2007). Divided attention impairs human motor adaptation but not feedback control. *Journal of Neurophysiology*, *98*(1), 317–326.

Tsay, J. S., Avraham, G., Kim, H. E., Parvin, D. E., Wang, Z., & Ivry, R. B. (2020). The Effect of Visual Uncertainty on Implicit Motor Adaptation. *Journal of Neurophysiology*. doi:10.1152/jn.00493.2020

Tsay, J. S., Ivry, R. B., Lee, A., & Avraham, G. (2021). Moving outside the lab: The viability of conducting sensorimotor learning studies online. *Neurons, Behavior, Data Analysis, and Theory*. doi:10.51628/001c.26985

Vandevoorde, K., & Orban de Xivry, J.-J. (2019). Internal model recalibration does not deteriorate with age while motor adaptation does. *Neurobiology of Aging*, *80*, 138–153.

Wang, T. S. L., & Song, J.-H. (2017). Impaired visuomotor generalization by inconsistent attentional contexts. *Journal of Neurophysiology*, *118*(3), 1709–1719.

Wei, K., & Körding, K. (2009). Relevance of error: what drives motor adaptation? *Journal of Neurophysiology*, *101*(2), 655–664.

Wei, K., & Körding, K. (2010). Uncertainty of feedback and state estimation determines the speed of motor adaptation. *Frontiers in Computational Neuroscience*, *4*, 11.

Zimmet, A. M., Cao, D., Bastian, A. J., & Cowan, N. J. (2020). Cerebellar patients have intact feedback control that can be leveraged to improve reaching. *ELife*, *9*. doi:10.7554/eLife.53246
