# Supplementary material for: Interactions between sensory prediction error and task error during implicit motor learning
Source: PLoS Comput Biol. 2022 Mar 23;18(3):e1010005. doi: 10.1371/journal.pcbi.1010005 (PMC8979451; doi:10.1371/journal.pcbi.1010005)
Supplement: Attachment — Submitted filename: TJ_RevisionLetter_12Feb2022_ToSubmit.docx [file pcbi.1010005.s006.docx]

Dear Dr. Tsay,

Thank you very much for submitting your manuscript "Interactions between sensory prediction error and task error during implicit motor learning" for consideration at PLOS Computational Biology. As with all papers reviewed by the journal, your manuscript was reviewed by members of the editorial board and by several independent reviewers. The reviewers appreciated the attention to an important topic. Based on the reviews, we are likely to accept this manuscript for publication, providing that you modify the manuscript according to the review recommendations. To be clear, these are really very minor revisions that we request, especially addressing the extra detail for the new experiment.

 Please prepare and submit your revised manuscript within 30 days. If you anticipate any delay, please let us know the expected resubmission date by replying to this email.

 When you are ready to resubmit, please upload the following:

 [1] A letter containing a detailed list of your responses to all review comments, and a description of the changes you have made in the manuscript. Please note while forming your response, if your article is accepted, you may have the opportunity to make the peer review history publicly available. The record will include editor decision letters (with reviews) and your responses to reviewer comments. If eligible, we will contact you to opt in or out

[2] Two versions of the revised manuscript: one with either highlights or tracked changes denoting where the text has been changed; the other a clean version (uploaded as the manuscript file).

Important additional instructions are given below your reviewer comments.

Thank you again for your submission to our journal. We hope that our editorial process has been constructive so far, and we welcome your feedback at any time. Please don't hesitate to contact us if you have any questions or comments.

Sincerely,

Aldo A Faisal

Associate Editor

PLOS Computational Biology

Wolfgang Einhäuser

Deputy Editor

PLOS Computational Biology

***********************

Reviewer's Responses to Questions

Reviewer #1: The revision has successfully addressed most concerns that I raised. In particular, the newly added experiment with a random TE but without SPE is a nice add-on for supporting the conclusions. However, this new experiment is not described in the revision; only a result figure is presented as Fig S1 (note the figure is not in the redlined manuscript either). As this experiment is critical for the main conclusion, I suggest including it in the main text. As I do not know the details of the experiment, I assume that the participants were instructed to ignore the random target jumps. Did they?

Based on the reviewer’s suggestion, we have moved the original material from Fig S1 in the first revision to now be part of the main manuscript. It is presented as Exp 1B and we summarize the key findings in the Results section (lines 119-131) and experimental details in the Methods section (lines 553 – 537 of the clean copy of the manuscript). We appreciate the reviewer’s prod to make this change: Not only is it important to provide methodological information, but Fig 2 now features a nice replication of some key results (e.g., absence of learning from TE-only). Note the changes here required us to restructure some figures, with Figure 2 now summarizing the results from Exp 1A, B and Figure 3 summarizing the results from Exp 2.

We want to point out a key difference between in-person (Exp 1A) and online (Exp 1B) experiments: As can be seen in Figure 2, implicit recalibration was greater in the in-person experiment compared to the online experiment for the smaller errors (±4° SPE + TE). We discuss potential reasons for this and other differences between the two contexts in a Supplemental Discussion titled, “Similarities and differences between in-person and online experiments.”

With this new experiment, it becomes clear that we need to interpret the findings more cautiously than now. The current conclusion is strong, i.e., target error alone cannot support implicit recalibration/learning. But I would suggest concluding this effect with its associated conditions. 1) Are the target jump findings generalizable to all learning based on target error? Saccadic adaptation is a similar implicit adaptation paradigm with a target jump in the middle of the movement, leading to trial-by-trial adaptation. Instead of ignoring the target jump, the instruction for saccadic adaptation is to make saccades to the target. How can we reconcile the discrepancy between the present study and saccadic adaptation? Can we explain it away by saying saccadic adaptation is not implicit, or their target error is task-contingent, but here it is not?

As noted by the reviewer, one key difference between a typical saccade adaptation task and our task relates to how participants are instructed to respond to target jumps. In the former, task error is task-relevant (or task-contingent) with the participant instructed to look at the target; in contrast, task error is not task-relevant in our task in the sense that participants were instructed to ignore the target jump and reach directly to the original target location. As such, it is possible that task error alone may be sufficient to drive learning when it is relevant to the goals of the task. Indeed, Ranjan and Smith (2020) instructed their participants to “hit the target” (meaning an adjustment was encouraged after the target jump) and observed adaptation in response to TE alone (Ranjan & Smith, 2020).

Note that we do not think the explicit/implicit distinction is key here: We assume that learning is implicit in “standard” saccade adaptation tasks, as well as in the Ranjan and Smith study. For saccade adaptation, the task jump typically occurs during the initial saccade and is outside awareness due to “saccade blindness.” One study that is very relevant to this issue is Cameron et al. (2010). Participants were instructed to saccade and reach to a target presented in the periphery. For one group, the target was jumped intra-saccadically (and thus remained outside awareness); for the other group, the target was jumped after the eye and reaching movement had terminated (and, as such, participants were aware of the perturbation). Interestingly, there was robust adaptation in the former and only weak adaptation in the latter. Thus, here is a case where adaptation was limited to the condition in which participants were unaware of the perturbation.

In sum, while we think task-relevance and awareness of the target displacement impacts the implicit response to the induced error, we also recognize that future studies are required to sort out their relative importance.

We address these issues in the revised Discussion section, Lines 347 - 368.

2) The zero slope observed in the new experiment is based on a fitting over a wide range of target errors (-16 to 16 degrees). Interestingly, there is a small negative slope if only the three small errors are included (-4, 0, and 4 degrees). If we assume the 16-degree jump is more explicit, but the 4-degree jump is more implicit, it appears that the target jump can lead to “pure” implicit learning. Of course, I would love to see the regression results for these small jumps.

We followed the reviewer’s suggestion and computed the slope coefficients using only the small task error (-4, 0, +4) conditions. None of the slopes from the four experiments were significantly different than zero (all 95% CIs included zero); thus, in the current design, we failed to find evidence supportive of the hypothesis that small target jumps can result in implicit adaptation. We comment on this post-hoc analysis in Lines 130 - 131, as well as in new Supplemental Table S2.

Table S2: TE-only fails to elicit implicit recalibration in response to small target displacements. The mean slope value and its 95% confidence interval were computed using only target jumps between ±4° for Experiments 1 & 2A and ±8° for Experiment 2B.

| Experiment | Mean slope [95% CI] | P value |
| --- | --- | --- |
| 1a | -0.02 [-0.15 0.10] | 0.72 |
| 1b | -0.05 [-0.12 0.03] | 0.20 |
| 2a | 0.01 [-0.06 0.07] | 0.62 |
| 2b | -0.02 [-0.05 0.01] | 0.72 |

3) what if the target jump is not presented in a fully random fashion but, say, in mini-blocks? If it is purely random, the nervous system might be better off aiming for the middle and thus completely ignoring the target error. That’s being said, should the current conclusion about zero effect of TE on implicit learning be limited to random trial-by-trial learning? Relatedly, the discussion about the random vs. block is not precise in Discussion (Line321). None of the referenced studies support the conclusion that “TE in the absence of SPE failed to elicit implicit recalibration” with a block design. This is still an unexplored issue.

We respectfully disagree with the reviewer on this point. The results from our trial-by-trial design are complemented by a recent preprint that used a blocked design to assess the effect of target jumps in the absence of SPE (Oza et al., 2020). We have pasted a figure from their preprint below. As can be seen, there was no evidence of implicit recalibration in response to either consistent 10° or 20° target jumps. (We cite this preprint in the Discussion.) We also note that the attenuating effects of jump-to (i.e., target jumping to a clamped cursor; SPE-only condition) in Exps 2-4 from our study were also seen in the blocked design used in Leow et al (Leow et al., 2018, 2020).

In summary, we believe that the pattern observed with our trial-by-trial design converges with other work using blocked designs.

Based on the reviewer’s comment, we have revised the manuscript to specify which studies used a SPE-only blocked design and which used a TE-only blocked design (Lines 320 - 323): “These observations obtained here using a trial-by-trial design are consistent with previous studies that have used a blocked design, in which either SPE-only trials (Kim et al., 2019; Leow et al., 2018) or TE-only trials (Oza, Kumar, & Mutha, 2020) remained invariant for an entire block.”

Line699: the reference is not in the right format. Please check other places too.

The references have been fixed.

Reviewer #2: I thank the authors for their careful consideration of the comments/suggestions raised in the first round of reviews. The paper has been greatly improved. I have no further comments at this time.

**Have the authors made all data and (if applicable) computational code underlying the findings in their manuscript fully available?**
The [PLOS Data policy](https://journals.plos.org/ploscompbiol/s/materials-and-software-sharing) requires authors to make all data and code underlying the findings described in their manuscript fully available without restriction, with rare exception (please refer to the Data Availability Statement in the manuscript PDF file). The data and code should be provided as part of the manuscript or its supporting information, or deposited to a public repository. For example, in addition to summary statistics, the data points behind means, medians and variance measures should be available. If there are restrictions on publicly sharing data or code —e.g. participant privacy or use of data from a third party—those must be specified.

Reviewer #1: **No:** they said the data will be available by request, but the model codes are not shared.

Both the data and code to reproduce the figures are now published on GitHub: https://github.com/xiaotsay2015/target_jump

Reviewer #2: Yes

**References**

Leow, L.-A., Marinovic, W., de Rugy, A., & Carroll, T. J. (2018). Task errors contribute to implicit aftereffects in sensorimotor adaptation. *The European Journal of Neuroscience*, *48*(11), 3397–3409.

Leow, L.-A., Marinovic, W., de Rugy, A., & Carroll, T. J. (2020). Task Errors Drive Memories That Improve Sensorimotor Adaptation. In *J. Neurosci.* (Issue 15, pp. 3075–3088). https://doi.org/10.1523/JNEUROSCI.1506-19.2020

Oza, A., Kumar, A., & Mutha, P. K. (2020). Task Errors Do Not Induce Implicit Sensorimotor Learning. In *Cold Spring Harbor Laboratory* (p. 2020.11.13.381285). https://doi.org/10.1101/2020.11.13.381285

Ranjan, T., & Smith, M. (2020, October 21). Implicit motor adaptation is driven by motor performance prediction error rather than sensory prediction error. *Advances in Motor Control and Motor Learning*.
